# Levels of Lead, Copper, and Zinc in Cabbage (*Brassica oleracea* sp.) and Lettuce (*Lactuca sativa* sp.) Grown on Soil Amended with Sewage Sludge

**DOI:** 10.1155/2021/8386218

**Published:** 2021-04-01

**Authors:** Bernard Fei-Baffoe, Jeffery Amo-Asare, Alhassan Sulemana, Kodwo Miezah

**Affiliations:** Department of Environmental Science, Kwame Nkrumah University of Science and Technology, Kumasi, Ghana

## Abstract

The study focused on levels of selected heavy metals (Pb, Zn, and Cu) in sewage sludge, soil, and in lettuce and cabbage grown on sewage sludge amended soil. Also, the effect of sewage sludge on soil properties (pH, soil organic matter, and soil conductivity) was studied. Three treatments were used based on nitrogen application rates of the sewage sludge: 100, 150, and 200 kg N/ha for lettuce while 160, 210, and 260 kg N/ha for cabbage. A pot experiment was conducted with pots arranged in a completely randomized design and under local climatic conditions. The study revealed that soil organic matter content and conductivity increased significantly with increasing nitrogen application rates. Levels of heavy metals in the soil increased significantly with increasing application rates. The controls for both plants recorded the lowest heavy metal uptake. Cabbage had an uptake of 0.48 **±** 0.13, 1.36 **±** 0.23, and 2.60 **±** 0.29 mg/kg for Pb, Zn, and Cu, respectively, while lettuce had 0.34 **±** 0.19, 1.35 **±** 0.31, and 2.30 **±** 0.14 mg/kg uptake for Pb, Zn, and Cu, respectively. Highest metal uptake was recorded at the highest application rate in both plants (0.66 **±** 0.17, 2.66 **±** 0.09, and 4.33 **±** 0.14 mg/kg for Pb, Zn, and Cu, respectively, for cabbage and 0.54 **±** 0.01, 2.24 **±** 0.17, and 3.88 **±** 0.19 mg/kg of Pb, Zn, and Cu, respectively, for lettuce). The uptake of Zn and Cu was significant, while Pb uptake was insignificant for both plants. Yields increased significantly with increasing application rates. The study provides information on yield enhancement resulting from cultivating plants on soil amended with sewage sludge and the associated health risk implication.

## 1. Introduction

One major challenge to agricultural production especially in developing countries is excessive nutrients loss. This challenge arises mainly from anthropogenic sources such as bad farming practices, industrial activities, and environmental pollution. Large areas of sub-Saharan Africa are affected by nutrient depletion [1]. For sub-Sahara Africa as a whole, nutrient depletion as posited by Choi and Yun [2], in 2003, accounts for about 7% of the agricultural gross domestic product of both crop and livestock production. This amounts to an annual cost of approximately US$32 per farm household or about US$20 for each hectare of arable land. With increasing population and the associated demand in food supply, the situation is expected to worsen. As a result of this, many regions of the world are in urgent need for greater nutrient inputs to support food production. This has prompted farmers to amend their soil with organic and inorganic fertilizers in order to enhance plant growth and increase crop yield. Sewage sludge as a form of organic fertilizer has been used by farmers to enrich the nutrient levels of soil and to increase crop yield.

The benefits of sewage sludge application in agricultural production in India were reviewed by Saha et al. [3]. The review presents the available information on various aspects of land application of municipal sewage sludge on crop yield and soil fertility. Kirchmann et al. [4] studied on the agricultural use of sewage sludge, and their findings indicate that the microbial biomass in soils was stressed until about 1990 by metal addition with sewage sludge. Thereafter, the microbial population recovered to its normal size due to lower metal loads, and the proportion of soil organic carbon reached a similar magnitude as in nonmetal-polluted soil. Other similar studies have also reported on the benefits of using sewage sludge in agricultural production [5–7].

Conversely, the spreading of sewage sludge on agricultural land has been known to result in increased levels of contaminants in soil. Lamastra et al. [8] assessed the suitability of sewage sludge as soil fertilizer in Northern Italy and found that most samples analyzed contained contaminants such as nonylphenol, nonylphenol ethoxylates, and phthalates, but their levels were within the EU permissible limits. Depending upon the origin, sewage sludge often contains very high amount of potentially toxic heavy metals, and as such, their excessive use for a longer period increases metal bioavailability in soil and ultimately causes food chain contamination [3]. These heavy metals are transferable, not biodegradable, and at some levels, they become toxic and tend to accumulate along the food chain [9, 10]. There is no much information on the characteristics of sewage sludge as well as suitability studies for agricultural use, particularly in developing countries.

There has been a surge in the use of sewage sludge for agricultural production. For instance, the Kwame Nkrumah University of Science and Technology (KNUST) Waste Treatment Plant produces huge tons of sewage sludge. Farmers within the catchment of KNUST use the untreated sewage sludge for cultivation of food crops. Vegetables, especially the leafy ones, have been known to accumulate high amounts of metals even in the presence of high levels of plant nutrients [11]. This as a result puts the health of consumers of such food crops at risk. There is, however, dearth of information on the levels of heavy metals in vegetables grown on soil amended with sewage sludge. It is against this background that the current study is conducted to determine the levels of some selected heavy metals (copper, zinc, and lead) in both cabbage (*Brassica oleracea*) heads and lettuce (*Lactuca sativa*) leaves grown on soil amended with sewage sludge at different application rates. The three heavy metals were selected for the study, since they are among the commonly reported pollutants from the use of sewage sludge for agricultural production and their potential toxicity to humans. The specific objectives of the study included assessing the effect of sewage sludge on soil properties and nutrient/heavy metal levels in lettuce and cabbage grown on sewage sludge amended soil.

## 2. Materials and Methods

### 2.1. Study Site and Sampling

The study was conducted at a selected cabbage farm located at Chirapatre in Kumasi, Ashanti Region of Ghana, with climatic conditions as follows; temperature ranges between 21.5°C and 30.7°C, average humidity about 84% at 0900 GMT and 60% at 1500 GMT, and maximum rainfall as 214.3 mm in June and minimum rainfall as 165.2 mm in September. Sewage sludge samples were obtained from the KNUST waste treatment plant and air dried within a period of two weeks at room temperature. The sludge at the time of sampling was three months old from the treatment process. The soil at the study site was treated with the sewage sludge. The treatments depended on application rates of the sludge based on recommended nitrogen levels: 160 kg N/ha, 210 kg N/ha, 260 kg N/ha, and 0 kg N/ha (control) for cabbage and 100 kg N/ha, 150 kg N/ha, 200 kg N/ha, and 0 kg N/ha (control) for lettuce [12–14]. The corresponding amounts of sludge (mg) for each of the treatments for the two plants (Table 1) were estimated using equation (1) developed by Cooperative Extension Service, University of Purdue, Indiana (https://www.extension.purdue.edu/extmedia/AY/AY-277.html). The treatments were applied to the experimental soils one month before plant cultivation. The treated soils (undisturbed sandy-loam) were collected at a depth of 5.0–8.0 cm, thoroughly homogenized and transferred to the experimental pots in a completely randomized design with three replications for each plant.

(1) $$\begin{array}{c} \text{Amount of sludge }\left(\text{mg}\right)=\frac{\text{AR }\left(\text{kg}-N/\text{ha}\right)\times \text{ AP }\left(\text{ha}\right)}{\text{NL }\left(\%\right)},\; \end{array}$$

where AR is the application rates of crops, i.e., 100, 150, and 200 kg N/ha for lettuce, while 160, 210, and 260 kg N/ha for cabbage; AP is the area of experimental pot = 42 by 28 cm = 1176 cm^2^ = 0.00001176 ha; NL refers to the nitrogen (total) level of the sludge = 3.43%.

### 2.2. Cabbage and Lettuce Cultivation

The seeds of cabbage and lettuce were nursed for four weeks and then randomly transplanted into the various experimental pots. One seedling was sown on each pot for cabbage, while two seedlings were sown on each pot for lettuce. The soils were stirred to enhance aeration, and garden fork was used to control weeds. Insecticides and weedicides were not applied to the experimental setup to avoid heavy metal contamination. Tap water was used to irrigate the plants twice daily. Lettuce plants were harvested on the third month after transplanting and cabbage plants harvested on the fourth month after transplanting.

### 2.3. Soil and Plant Analyses

#### 2.3.1. Determination of Soil Physicochemical Properties

Soil pH and conductivity were measured using a pH and conductivity meter (HQ40D, Hach). The pH meter was calibrated with buffer solutions at pH 4 and 7 prepared from citric acid and monopotassium phosphate, respectively, with potassium hydroxide. The conductivity of the prepared solution of the soil samples was measured after calibration with potassium chloride at 20°C. The organic matter content of the soil samples was determined by loss of weight on the ignition method as explained by the FAO [15]. A measured soil sample, 6.0 g, was dried in an oven at 105°C. It was placed in a muffle furnace, and the temperature was gradually increased to 440°C. The nitrogen content of the soil samples was determined using the Kjeldahl method as described by the FAO [15] and reviewed by Saez-Plaza et al. [16]. Sulphuric acid-digested soil was buffered with salicylate for colorimetric detection. The nitrogen content (mg/kg) determined was expressed as percentage. Phosphorus content was analyzed by Bray's method (No. 1). Vanado molybdate reagent was used for calorimetric detection. The percentage transmittance (% *T*) was measured at 430 nm, the absorbance was determined, and *P* content was obtained from a standard curve.

#### 2.3.2. Heavy Metals Determination

The test solution prepared from 5 ml of 8 N HCl was analyzed for the concentrations of lead, copper, and zinc in soil, sludge, and amended samples using atomic absorption spectrophotometry (AAS). The instrument was first calibrated with blank and working solutions of the various metals determined. The collected vegetable samples were washed with distilled water to remove dust particles. The samples were then cut to separate the roots, stems, and leaves using a knife. The leaves of lettuce and heads of cabbage were air dried and then placed in a dehydrator at 80°C for 2-3 days. An ignited residue was moistened with 2 ml distilled water, and 5 ml of 8 N HCl was added and filtered through Whatman No. 42 filter. The AAS was then used to determine the levels of copper, lead, and zinc in the lettuce and cabbage plants.

#### 2.3.3. Method Validation and Quality Control

Validity of the analytical methods used was based on control measures employed in the standard measurements employed and as per the measures from Prichard and Barwick [17]. All the instrumental measurements were conducted with seven prepared standard solutions of different concentrations and the blank before applying to the test solutions. Spiking solutions and methods blank were made to follow the same digestion process as the actual test samples for validation of limit of detection, quantification of the equipment, as well as precision and accuracy of the results.

### 2.4. Determination of Cabbage and Lettuce Yield

The average fresh weight of the two lettuce plants grown in each pot was determined with a Mettler balance. The weight of each fresh head of the cabbage in each pot was also determined with the Mettler balance.

### 2.5. Statistical Analysis

All analyses were carried out in three replicates per sample. Data were reported as mean ± standard error. One-way analysis of variance was used to determine the significant difference between treatments considering a level of significance of less than 5%. Multiple comparison using least significance difference was conducted to determine where specific differences occurred.

## 3. Results and Discussion

### 3.1. Background Concentration

The background concentrations of sludge and soil used in the study are presented in Table 2. pH of soil was within the recommended soil pH for plant growth (5.5–7.0), while the sludge pH was within the acidic range of the pH scale. The nutrient levels (phosphorus and nitrogen), soil conductivity, and organic matter contents in sewage sludge were higher than the levels in topsoil. The high nutrient levels recorded in the sewage sludge conform to the findings of previous studies [5, 18, 19]. Lower heavy metal levels were present in the topsoil as compared to the sewage sludge for all the selected heavy metals. The presence of heavy metals in the sewage sludge accord with several research works which reported the presence of heavy metals in sewage sludge as a major setback to its application in agriculture [20–22]. Levels of metals in the soil samples were all within the EU directives of acceptable limits of heavy metals in soil: Pb (50–300 mg/kg), Cu (50–140 mg/kg), and Zn (150–300 mg/kg). The levels of metals in sewage sludge were also within the EU directives of acceptable limits of heavy metals in sewage sludge: Pb (750–1200 mg/kg), Cu (1,000–1,750 mg/kg), and Zn (2500–4000 mg/kg).

### 3.2. Effects of Sewage Sludge on Soil after Applying Treatments

**Figure 1:**
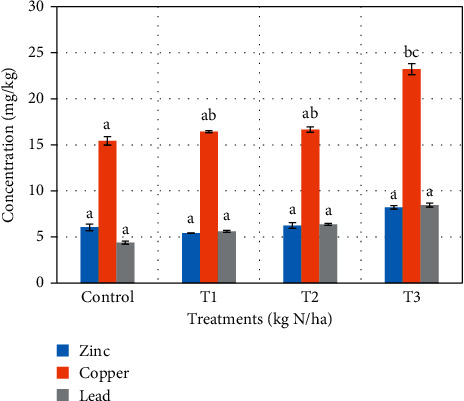
Heavy metals uptake in soil after sewage sludge application (cabbage heads).

**Figure 2:**
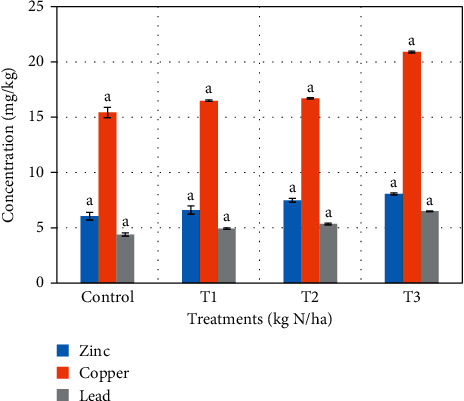
Heavy metals uptake in soil after sewage sludge application (lettuce leaves).

Tables 3 and 4 show the effect of sewage sludge on soil after treatments application for cabbage and lettuce. Increasing application rates increased the pH slightly, but the differences were not statistically significant. Sewage sludge has been known to alter the pH of soil. Kazi et al. [23] reported that pH of soil was insignificantly affected after amending with sewage sludge. An increase in soil pH has been reported in soils amended with sewage sludge [24], and lowering of soil pH is also reported [25]. The changes in soil pH have been associated with the calcium carbonate content of sludge and acid production during sludge decomposition [24]. Increasing application rates increased the organic matter contents as well as conductivity of the soil. The increase of soil conductivity and organic matter content was statistically significant (*p* < 0.05). From Tables 3 and 4, specific differences occurred between the control (*T*_c_) and the three treatments (*T*_1_–*T*_3_) for soils used for cabbage and lettuce cultivations, respectively. Ramulu and Sree [26] reported an increase in the organic matter as a result of sewage sludge application. The increase in soil conductivity has been attributed to organic matter which contains metallic salts [27, 28]. Levels of heavy metals in soils for cabbage and lettuce increased with increasing application rates (Figures 1 and 2). Although there was increase in heavy metal levels after sewage sludge application, statistically significant difference occurred only between the *T*_c_ and *T*_3_ for copper (*p*=0.032) in Figure 1. Several other studies have reported on the increase in heavy metal levels as a result of sewage sludge application [9, 10, 29, 30].

### 3.3. Heavy Metal Levels in Cabbage and Lettuce Grown on Various Treatments

**Figure 3:**
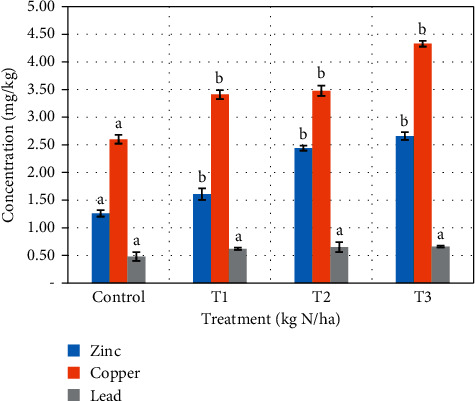
Heavy metals uptake in cabbage after cultivation on sewage-amended soil.

**Figure 4:**
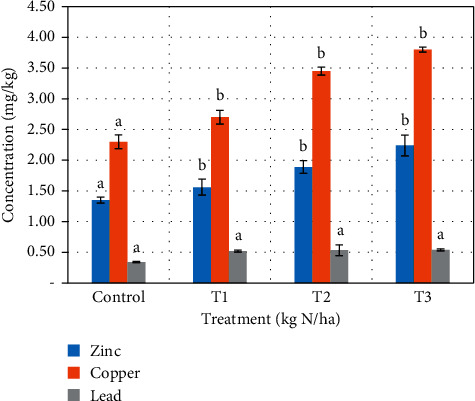
Heavy metals uptake in lettuce after cultivation on sewage-amended soil.

The controls for both plants recorded the lowest levels of heavy metals uptake (0.48 ± 0.13, 1.36 ± 0.23, and 2.60 ± 0.29 mg\kg for lead, zinc, and copper, respectively) in cabbage (Figure 3) and (0.34 ± 0.19, 1.35 ± 0.31, and 2.30 ± 0.14 mg\kg for lead, zinc, and copper, respectively) in lettuce (Figure 4). The highest levels were obtained from the highest application rates (0.66 ± 0.17, 2.66 ± 0.09, and 4.33 ± 0.14 for lead, zinc, and copper, respectively) for cabbage and (0.54 ± 0.01, 2.24 ± 0.17, and 3.88 ± 0.19 mg/kg for lead, zinc, and copper, respectively) for lettuce. Uptake of all heavy metals was statistically significant (*p* < 0.05) between the controls and the three treatments, except lead which was not significant in both plants.

Many factors have been attributed to the cause of uptake of heavy metals by vegetables. Liu et al. [31] and Bi et al. [32] reported high metal uptake in vegetables because of higher levels in background soil as a result of industrial activities. Other studies have reported similar findings of higher heavy metal uptake because of higher levels in sewage sludge [21, 22, 33, 34]. Contrary to these findings, McBride et al. [35] and Ok et al. [36] recorded lower metal uptake as a result of higher organic matter content and soil conductivity which immobilized heavy metals. Higher phosphates in soil have been reported to immobilize lead thereby reducing the bioavailability of lead [37]. The uptake of metals by plants can be attributed to the pH of the soil. pH (<6) has been reported to enhance leaching of heavy metals, making them available for plants uptake [23]. Demirezen and Ahmet [38] and Sharma et al. [39] have also attributed the uptake of heavy metals to the wastewater used for irrigation. The levels of metals recorded in the cabbage and lettuce used in this study were all below the WHO safe limit of the selected heavy metals in vegetables for human consumption, except Pb which recorded values greater than 0.3 mg/kg.

### 3.4. Effect of Sewage Sludge on Cabbage and Lettuce Yield

**Figure 5:**
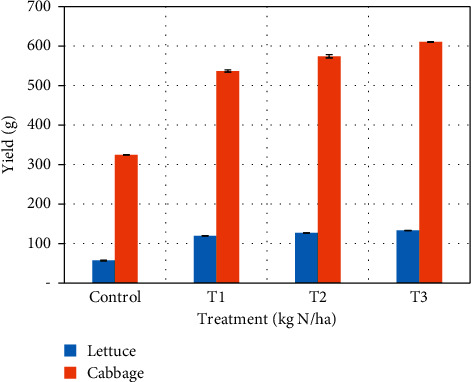
Mean values of cabbage and lettuce yields after cultivation.

The yields of cabbage and lettuce increased as nitrogen application rates increased (Figure 5). Lower yields were recorded in the control for both plants (324.50 ± 0.264 g for cabbage and 56.75 ± 0.99 g for lettuce). Yields of the other treatments (536.75 ± 2.87–610.50 ± 0.93 g) except control for cabbage were found to be within the market yield, while the yields of treatments for lettuce (119.58 ± 0.18–132.75 ± 0.19 g) including control were all within market yield. The increase in yields for both plants was statistically significant (*p* < 0.05). The increase in yield as a result of sewage sludge application conforms to works by researchers that indicate that sewage sludge is similar to commercial fertilizers and has the potential of increasing the yield of crops and producing high-quality crops [40, 41]. The increase in yield as a result of sewage sludge is attributed to its high nutrients content which positively affects the soil structure, improve soil aeration, and enhance the activities of living organisms within the soil.

## 4. Conclusion

This study showed that heavy metals (copper, lead, and zinc) were present in the sewage sludge from the KNUST treatment plant as well as the top soil used. The levels of metals in both sludge and soil were below the acceptable limits of EU directives. Application of sewage sludge to soil improved the soil organic matter content and increased soil conductivity because of high organic matter content and metallic ions of the sewage sludge. The sludge pH was within the acidic range but had no significant influence on soil pH. Applying sewage sludge increased the levels of heavy metals significantly in the soil but were below the EU acceptable limits. This study revealed that uptake of metals by lettuce and cabbage increased as the application rates of sewage sludge increased. The uptake of copper and zinc were significant for both plants, while uptake of lead was insignificant for both plants. The levels of metals in the lettuce and cabbage were all below the WHO safe limits of metals in plants for human consumption, except Pb. Cabbage and lettuce yield increased with increasing application rates of sewage sludge. The yield increased at the highest application rates, while the lowest yields were recorded at the control. This shows that the use of KNUST treatment sludge for cultivation of cabbage and lettuce can improve crop yields but with increased heavy metals uptake, especially copper and zinc which are quite below acceptable limits. Treatment of the sludge is required before use for agricultural production to avert the potential risk to human health.

## Figures and Tables

**Table 1:** Nitrogen application rates and corresponding amount of sludge used as treatment.

Code	Cabbage		Lettuce	
	Application rate (kg N/ha)	Sludge amount (mg)	Application rate (kg N/ha)	Sludge amount (mg)
*T*_1_	160	5.49	100	3.40
*T*_2_	210	7.20	150	5.14
*T*_3_	260	8.90	200	6.86
*T*_c_	0	0.00	0	0.00

*T*_c_, control without sludge amendment.

**Table 2:** Mean values of background concentration of sludge and topsoil.

Parameter	Sludge	Soil
pH	4.72 ± 0.27	6.55 ± 0.16
Phosphorus (ppm)	21.81 ± 2.57	5.22 ± 0.43
Nitrogen (%)	3.48 ± 0.12	0.15 ± 0.03
Organic matter (%)	65.63 ± 1.63	6.93 ± 0.40
Zinc (mg/kg)	40.77 ± 0.36	6.05 ± 0.13
Copper (mg/kg)	53.10 ± 0.22	15.43 ± 0.38
Lead (mg/kg)	24.10 ± 0.13	4.39 ± 0.17
Conductivity (*μ*s/cm)	1365.33 ± 21.57	257 ± 9.54

**Table 3:** Mean values of selected soil properties after sludge application for cabbage cultivation.

Parameters	*T*_c_	*T*_1_ (160 kg/ha)	*T*_2_ (210 kg/ha)	*T*_3_ (260 kg/ha)
pH	6.55 ± 0.33	6.69 ± 0.05	6.71 ± 0.15	6.75 ± 0.06
Soil conductivity (*μ*s/cm)	257 ± 13.16^a^	346.75 ± 17.82^b^	365.25 ± 5.68^b^	382.25 ± 13.30^b^
Organic matter content (%)	6.93 ± 0.08^a^	17.435 ± 0.82^b^	20.475 ± 0.59^b^	24.425 ± 0.67^b^

*T*_1_, treatment 1, *T*_2_, treatment 2; *T*_3_, treatment 3. Different letters (superscripts) indicate statistically significant differences (*p* < 0.05).

**Table 4:** Mean values of selected soil properties after sludge application for lettuce cultivation.

Parameters	*T*_c_	*T*_1_ (160 kg/ha)	*T*_2_ (210 kg/ha)	*T*_3_ (260 kg/ha)
pH	6.55 ± 0.33	6.71 ± 0.10	6.68 ± 0.13	6.74 ± 0.11
Soil conductivity (*μ*s/cm)	257 ± 13.16^a^	331 ± 19.77^b^	343.75 ± 19.21^b^	364.75 ± 10.31^b^
Organic matter content (%)	6.93 ± 0.08^a^	14.675 ± 1.07^b^	16.5 ± 0.28^b^	18.87 ± 0.79^b^

*T*_1,_ treatment 1; *T*_2_, treatment 2; *T*_3_, treatment 3. Different letters (superscripts) indicate statistically significant differences (*p* < 0.05).

## Data Availability

The data used to support the findings of this study are available from the corresponding author upon request.
